# Prevalence of Occult Infection by Hepatitis B Virus in Liver Biopsies in Clinical Practice

**DOI:** 10.3390/v18101114

**Published:** 2026-10-08

**Authors:** Diana Horta, Angels Ramos, Raimon Rifà, Carme Loras, Marcos Jimenez-Sena, Agnes Raga, Maria Esteve-Comas

**Affiliations:** 1 Department of Gastroenterology and Hepatology, Hospital Universitari Mútua Terrassa, Universitat de Barcelona, 08221 Terrassa, Spain; 2 Centro de Investigación Biomédica en Red de Enfermedades Hepáticas y Digestivas (CIBERehd), Instituto de Salud Carlos III, 28029 Madrid, Spain; 3 Department of Microbiology, Catlab, Parc Logístic de Salut, Viladecavalls, 08232 Barcelona, Spain

**Keywords:** hepatitis B, occult hepatitis B infection, polymerase chain reaction, liver biopsy, HBV-DNA

## Abstract

Occult hepatitis B infection (OBI) is defined by detectable hepatitis B virus (HBV) DNA in liver tissue in the absence of hepatitis B surface antigen (HBsAg) and remains insufficiently characterized in low-endemic European settings. This study aimed to determine the prevalence of OBI using direct HBV DNA detection in liver tissue in a real-world cohort of patients undergoing diagnostic liver biopsy. This observational study included consecutive HBsAg-negative patients who underwent liver biopsy between 2020 and 2023. HBV DNA in liver tissue was quantified by real-time PCR, and clinical and serological data were collected. A total of 86 patients were included. OBI was identified in 4 patients (4.7%). All OBI cases were anti-HBc-positive, and two were also anti-HBs-positive. No cases of seronegative OBI were detected. OBI was observed across different underlying liver disease etiologies, including viral, metabolic, autoimmune, and cholestatic conditions. In conclusion, OBI was infrequent in this cohort and consistently associated with anti-HBc seropositivity. These findings suggest that anti-HBc may help identify individuals with prior HBV exposure in whom occult infection can be detected in liver tissue in low-prevalence settings.

## 1. Introduction

Chronic hepatitis B virus (HBV) infection affects approximately 240 million people worldwide and is traditionally defined by the persistence of hepatitis B surface antigen (HBsAg) in serum for at least six months [1]. HBV infection displays a broad clinical spectrum, ranging from asymptomatic infection and self-limited acute hepatitis to chronic hepatitis, progressive fibrosis, cirrhosis, decompensated liver disease, and hepatocellular carcinoma (HCC). Importantly, liver injury in HBV infection is largely immune-mediated rather than directly cytopathic, reflecting the interaction between viral persistence and host immune responses. Even after apparent clinical recovery, HBV may persist in the liver due to the long-term stability of covalently closed circular DNA (cccDNA) within hepatocyte nuclei [2].

In recent years, growing attention has been focused on occult hepatitis B infection (OBI). OBI is characterized by the presence of HBV DNA in liver tissue, with undetectable HBsAg by current routine assays and variable detection of HBV DNA in serum [2]. OBI can be classified into seropositive and seronegative forms [3]. Seropositive OBI shows detectable serum anti-HBc with or without anti-HBs, while seronegative OBI, less common and accounting for approximately 20% of cases, lacks detectable anti-HBc and anti-HBs in serum [2,4]. Although serologically silent, OBI is clinically relevant because it may be transmitted through blood transfusion or organ transplantation, reactivate under conditions of immunosuppression, contribute to the progression of chronic liver disease, and play a role in hepatocarcinogenesis [4,5].

The biological basis of OBI is characterized by the long-term persistence of replication-competent HBV DNA in hepatocytes despite the absence of detectable HBsAg. This condition is thought to result from a marked suppression of viral replication and transcription, leading to very low levels of viral protein expression. Current evidence suggests that host-related factors, including immune-mediated control and epigenetic regulation of intrahepatic cccDNA, play a central role in maintaining this low replicative state [5]. Although mutations within the pre-S/S region may impair HBsAg production, secretion, or detection by conventional assays and contribute to occult infection in selected cases, these viral variants appear to account for only a minority of OBI cases [6,7].

Diagnosing OBI remains a clinical challenge due to the lack of standardized and widely validated detection methods. The gold standard is the detection of HBV DNA directly in liver tissue by sensitive PCR techniques. However, liver biopsy is an invasive procedure, limiting its routine use. Consequently, non-invasive markers, particularly serum HBV DNA, are commonly used to support the identification of OBI, although they have important limitations. Among these, total anti-HBc is the most informative serological marker and is widely used to screen for OBI, since it identifies previous exposure to HBV [2,5]. However, a positive result does not by itself demonstrate intrahepatic HBV persistence. Because viral replication remains strongly suppressed in OBI, serum HBV DNA is often intermittently detectable and, when present, usually found at very low levels (<200 IU/mL) [5]. Therefore, studies relying exclusively on serum markers may underestimate the true prevalence of occult infection [5].

The reported prevalence of OBI varies considerably according to the studied population, the biological samples analyzed, the diagnostic methods used, and HBV vaccination coverage [5]. Accurate diagnosis is crucial since OBI has been implicated in the progression of chronic liver diseases, cryptogenic cirrhosis, and hepatocellular carcinoma of unknown etiology [8].

Several studies have assessed the prevalence of OBI in diverse groups such as blood donors [9,10], healthy adults [11,12,13], HIV-infected individuals [14], hemodialysis patients [15,16], patients with cryptogenic liver disease or cirrhosis [17,18], and those with HCC of unknown origin [19,20,21]. However, these studies often rely on surrogate serum markers and lack a uniform definition of OBI.

Few studies have investigated OBI prevalence directly in liver tissue obtained via biopsy. For example, in a Brazilian cohort of liver transplant candidates, the prevalence of OBI detected by liver PCR was reported at 4.4% [22]. In patients with chronic hepatitis C virus (HCV) infection, studies have shown OBI prevalence in liver biopsies ranging from approximately 6% to 30% [8,23,24,25], varying by population and detection methods. A study from Morocco assessing patients with cryptogenic and HCV-related cirrhosis found an OBI prevalence of around 7% [26]. Similarly, research in Taiwan reported OBI presence in patients with hepatocellular carcinoma, highlighting the virus’s possible role in carcinogenesis [27].

Except for a limited number of studies from Italy evaluating intrahepatic HBV DNA in liver tissue from transplant recipients and HBsAg-negative individuals [12] and from morbidly obese patients undergoing bariatric surgery [28], data from European populations remain scarce and are generally based on small cohorts. Therefore, information regarding the prevalence of OBI in liver biopsies from routine clinical practice is still limited.

The aim of this study was to evaluate the prevalence of occult hepatitis B infection in patients undergoing liver biopsies at our center, reflecting routine clinical practice in our population.

## 2. Materials and Methods

### 2.1. Participants and Eligibility Criteria

This observational, single-center study was conducted at the Hepatology Department of Hospital Universitari Mútua Terrassa (Spain) and included consecutive adult outpatients with negative serum HBsAg who underwent liver biopsy between 2020 and 2023. Exclusion criteria were tumor-related liver biopsy and age under 18 years.

The study was approved by the institutional review board (TASDA_003) and complied with Good Clinical Practice guidelines and the Declaration of Helsinki. Written informed consent was obtained from all participants.

### 2.2. Data Collection

Upon study enrollment, demographic, clinical, laboratory, and imaging data were prospectively collected. Variables assessed included age, sex, ethnicity, alcohol consumption, etiology of liver disease, liver function tests, transient elastography results, and abdominal ultrasound findings. Serological HBV markers evaluated at baseline included HBsAg, anti-HBc, anti-HBs, and serum HBV DNA. Furthermore, considering the epidemiological background of the study population, it is important to note that a mass universal hepatitis B vaccination program targeting pre-adolescents in schools was implemented in Catalonia in 1991 [29]. As this program led to a dramatic decline in acute HBV infection incidence in the region, the HBV vaccination status of the patients included in our cohort was systematically recorded whenever available.

### 2.3. Liver Biopsy Procedure and Tissue Sampling

Liver biopsies were predominantly performed via a percutaneous ultrasound-guided approach using 16G needles (Tru-Cut, Bard Peripheral Vascular, Tempe, AZ, USA). However, depending on patient characteristics, clinical indications, and institutional procedural availability at the time of the biopsy, an endoscopic ultrasound-guided (EUS) approach was also utilized. EUS-guided biopsies were conducted using 19G cytohistological needles (EchoTip ProCore, Cook Medical, Bloomington, IN, USA). Regardless of the approach used, the quality and yield of the tissue were adequate for both diagnostic and research purposes. In addition to the tissue specimens routinely required for diagnostic histopathological evaluation, an extra liver tissue core was specifically collected for research purposes from all participating patients, following informed consent. Immediately after collection, this study-specific tissue specimen was frozen and stored at −80 °C until HBV DNA analysis.

### 2.4. Liver Tissue HBV DNA Quantification

Liver biopsy specimens were immediately stored at −80 °C after collection and maintained at this temperature until HBV DNA analysis. For each determination, up to 5 mg of frozen liver tissue was used. Tissue samples were homogenized by adding 180 μL of MagNA Pure 96 DNA Tissue Lysis Buffer and 20 μL of proteinase K (Roche Diagnostics, Basel, Switzerland), followed by overnight incubation at 37 °C to ensure complete tissue lysis. Subsequently, 180 μL of physiological saline was added to reach the minimum sample volume required for processing on the cobas^®^ 6800 System (Roche Diagnostics, Basel, Switzerland; software version 2.0).

HBV DNA extraction, amplification, and quantification were performed using the cobas^®^ HBV assay (Roche Diagnostics) according to the manufacturer’s instructions. The assay is a quantitative real-time PCR assay targeting highly conserved regions of the HBV pre-core and core genomic regions. The cobas^®^ HBV assay is commercially validated for HBV DNA quantification in human EDTA plasma and serum, but not for liver tissue. Therefore, in the present study, the assay was applied to liver biopsy specimens using a laboratory-adapted tissue lysis and sample preparation protocol. This approach is in line with the current recommendations, as no standardized liver HBV DNA assay with internal and external validation is yet available; real-time PCR, nested PCR, and droplet digital PCR have all been proposed as suitable approaches for intrahepatic HBV DNA detection [5]. Positive results were confirmed by repeat testing from the same tissue lysate. The assay has a lower limit of quantification of 10 IU/mL and an upper limit of quantification of 1.0 × 10^9^ IU/mL for the applicable validated sample matrices. For the purposes of this study, HBV DNA positivity was defined as a detectable and quantifiable HBV DNA result within the assay’s quantification range.

### 2.5. Definition of Occult Hepatitis B Infection

Occult hepatitis B infection (OBI) was defined as the presence of detectable HBV DNA in liver tissue in patients with negative serum HBsAg, regardless of anti-HBc or anti-HBs status.

### 2.6. Endpoints

The primary endpoint was to determine the prevalence of OBI in liver biopsies from HBsAg-negative patients. Secondary endpoints included the description of demographic and clinical characteristics of patients with OBI.

### 2.7. Statistical Analysis

Continuous variables are expressed as mean ± standard deviation (SD), and categorical variables as frequencies and percentages. Given the small number of OBI cases, only descriptive analyses were performed. Statistical analyses were performed using SPSS software (version 25.0; IBM Corp., Armonk, NY, USA).

## 3. Results

### 3.1. Baseline Characteristics

A flow chart for the study is provided in Figure 1. A total of 109 outpatients who underwent liver biopsy in routine clinical practice were initially identified. Of these, 101 patients agreed to participate in the study. Twelve patients were excluded due to predefined exclusion criteria, leaving 89 liver tissue samples available for HBV DNA PCR analysis. Three samples yielded invalid PCR results, resulting in a final study cohort of 86 patients.

Baseline characteristics are summarized in Table 1. Mean age was 49.8 ± 11.6 years, and 53.5% of patients were female. Most participants were Caucasian (91.9%), and liver cirrhosis was present in 10.5% of cases. The most frequent etiologies of liver disease were HCV infection (29.1%), MASLD (25.6%), autoimmune hepatitis (HAI) (23.3%), and primary biliary cholangitis (PBC) (15.1%). Anti-HBc and anti-HBs positivity were detected in 15.1% and 17.4% of patients, respectively, with 7.0% showing a combined anti-HBc and anti-HBs positive pattern. Documented HBV vaccination was present in 10 patients (11.6%), with a mean age of 31.9 ± 8.9 years among vaccinated individuals.

### 3.2. Prevalence of Occult HBV Infection

OBI was detected in 4 of 86 patients, resulting in an overall prevalence of 4.7%. All OBI-positive patients were anti-HBc-positive, corresponding to a prevalence of 30.8% (4/13) among individuals with serological evidence of previous HBV exposure. Two of the OBI-positive patients were also positive for anti-HBs antibodies. No cases of OBI were identified among the remaining 73 anti-HBc-negative patients. The underlying liver diseases among OBI-positive patients included HCV infection, MASLD, AIH, and PBC. None of the patients with OBI had liver cirrhosis at baseline. All four OBI-positive patients were of Spanish origin. The four OBI-positive patients are described individually in Table 2 to provide a detailed overview of their clinical, serological, and virological characteristics.

Serum HBV DNA was detectable in only 1 of the 4 OBI-positive patients, with a viral load of 25 IU/mL, whereas it was undetectable in the remaining 3 patients. Thus, 75% of the OBI-positive cases had undetectable HBV DNA in serum at the time of assessment.

## 4. Discussion

In this study, we evaluated the prevalence of OBI in a real-world cohort of consecutive patients undergoing liver biopsies in routine clinical practice. Using direct HBV DNA detection in liver tissue, OBI was identified in 4.7% of HBsAg-negative patients. Importantly, all OBI cases occurred in anti-HBc-positive individuals, and no OBI cases were detected in seronegative cases. In addition, OBI was detected in 30.8% (4/13) of anti-HBc-positive patients; however, given the limited number of exposed individuals, this observation should be interpreted as descriptive.

The prevalence of OBI reported in the literature is highly variable and strongly influenced by the studied population, underlying liver disease, HBV endemicity and, importantly, the diagnostic method [30]. In particular, studies based on serum markers and peripheral blood HBV DNA tend to report lower prevalence estimates in low-risk populations, such as blood donors, and higher rates in groups with previous HBV exposure or advanced liver disease, including patients with HCV infection, HIV infection, or cirrhosis [31,32,33,34,35]. This heterogeneity should be taken into account when interpreting prevalence data, as studies relying exclusively on serum-based testing may underestimate the true burden of intrahepatic HBV persistence.

In contrast, studies using liver tissue PCR, which remains the most sensitive method for detecting intrahepatic HBV persistence, have reported highly variable OBI prevalence estimates across different clinical settings [36]. In biopsy-based cohorts of patients with chronic liver disease, intrahepatic HBV DNA has been detected in approximately 22% of cases in studies from Iran [37]. Similarly, studies in specific liver disease contexts such as metabolic dysfunction-associated steatotic liver disease (MASLD) have also demonstrated detectable intrahepatic HBV DNA in a relevant subset of patients, supporting the concept that occult infection may coexist with diverse etiologies of chronic liver disease [28].

Lower prevalence rates are typically observed in unselected hepatology cohorts and in low-endemic settings. In this context, a study in Brazilian liver transplant recipients reported an OBI prevalence of approximately 4% based on liver tissue analysis [22], which is consistent with the low prevalence observed in our cohort, although differences in population and clinical context must be considered.

Direct comparisons between studies remain challenging due to substantial heterogeneity in study design, inclusion criteria, and laboratory methodologies. This variability highlights that OBI prevalence is highly context-dependent [5].

In our cohort, the observed prevalence of 4.7% should be interpreted within the specific epidemiological and clinical context of our study rather than directly compared with estimates from other biopsy-based studies. Our study included a consecutive, non-selected series of HBsAg-negative patients undergoing liver biopsy in routine clinical practice within a low-endemic European setting. Therefore, our cohort likely reflects a broader and less selected spectrum of liver diseases compared with studies enriched for patients with prior HBV exposure or specific high-risk conditions. Differences in patient selection and exclusion criteria may also contribute to the variability in reported prevalence across studies. Furthermore, this low-endemic context is largely attributable to the universal hepatitis B vaccination program introduced in Catalonia in 1991, which initially targeted pre-adolescents aged 11–12 years and was subsequently expanded to infants [29]. With the high coverage of this immunization strategy over more than three decades, the incidence of both overt HBV infection and OBI would be expected to decline in younger generations. In our cohort, the low proportion of documented vaccination is consistent with the fact that most participants were born before the implementation of the vaccination program. Although none of the OBI-positive patients had documented HBV vaccination, this observation should be interpreted cautiously given the small number of cases and the confounding effect of age.

Notably, all OBI-positive patients in our cohort were anti-HBc-positive, supporting the role of anti-HBc as a marker of previous HBV exposure [2,5]. Conversely, none of the 73 anti-HBc-negative patients had detectable intrahepatic HBV DNA, whereas serum HBV DNA was detectable in only one of the four OBI cases; in our low-endemic setting, total anti-HBc was therefore the most efficient available screening marker for OBI. This is consistent with the concept that OBI frequently represents a state of viral persistence after apparent serological resolution of infection, although alternative scenarios such as long-term low-level infection cannot be excluded [5].

In our cohort, 4 of 13 anti-HBc-positive patients (30.8%) had detectable intrahepatic HBV DNA. Although this proportion should be interpreted cautiously because of the small number of anti-HBc-positive individuals, it supports the need for larger studies specifically evaluating HBsAg-negative, anti-HBc-positive populations. Such studies could help better define the frequency and clinical significance of intrahepatic HBV persistence and identify patients who may benefit from further assessment for OBI.

Importantly, serum HBV DNA was detectable in only 1 of the 4 patients with OBI and was undetectable in the remaining 3 patients. Thus, 75% of OBI cases had no detectable HBV DNA in serum at the time of assessment. This finding is consistent with the low and potentially intermittent levels of circulating HBV DNA characteristic of OBI and highlights an important limitation of relying exclusively on serum HBV DNA to identify occult infection [5]. In this context, direct assessment of intrahepatic HBV DNA may increase the sensitivity for detecting viral persistence, particularly in patients in whom circulating HBV DNA is below the detection threshold of conventional serum-based assays. Nevertheless, these findings should be interpreted cautiously given the small number of OBI cases and the absence of longitudinal serum HBV DNA measurements.

In contrast, no cases of seronegative OBI were identified in our cohort. This form has been described in a minority of patients in previous studies, generally accounting for approximately 10–20% depending on cohort characteristics and assay sensitivity [2]. The absence of seronegative cases in our series may reflect the low prevalence of prior HBV exposure in our population.

OBI cases were distributed across different etiologies of liver disease, including viral, metabolic, autoimmune, and cholestatic conditions. This supports the concept that occult HBV infection may occur independently of the underlying cause of liver injury and may represent a persistent intrahepatic viral reservoir [38,39,40].

From a clinical perspective, OBI remains relevant due to its potential implications in disease progression [41], hepatocarcinogenesis [42,43,44,45], and HBV reactivation under immunosuppression [46,47]. Although none of the OBI-positive patients in our cohort had cirrhosis at baseline, previous studies have suggested that even low-level intrahepatic viral persistence may contribute to fibrogenesis and carcinogenic risk.

Several limitations should be acknowledged, including the small sample size, the low number of OBI cases, the single-center design, and the fact that serum HBV DNA was assessed at a single time point; therefore, intermittent or transient low-level viremia cannot be excluded in patients with undetectable serum HBV DNA. Sampling error is also inherent to any biopsy-based assessment of intrahepatic HBV DNA. A needle biopsy samples only a minute fraction of the organ, in the order of 1/50,000 of the total hepatic mass [48], and in OBI viral genomes are present at very low copy numbers and may be unevenly distributed among hepatocytes. A negative result therefore cannot formally exclude OBI, and the prevalence reported here should be regarded as a minimum estimate, as is the case for all biopsy-based OBI studies. In addition, the cobas^®^ HBV assay used for HBV DNA quantification is validated for plasma and serum but not for liver tissue. However, as no standardized liver HBV DNA assay with internal and external validation is currently available, real-time PCR-based approaches such as the one used in this study are considered an appropriate strategy [5]. Finally, HBV genotyping and intrahepatic cccDNA activity were not assessed, and anti-HBc was determined qualitatively; quantitative anti-HBc was not available in our cohort, and its potential value as a marker of the extent of previous HBV exposure deserves evaluation in future studies.

Despite these limitations, our study has several strengths. It is based on a consecutive real-world cohort of patients undergoing liver biopsy in routine clinical practice, reducing selection bias. In addition, OBI was assessed using direct HBV DNA detection in liver tissue, which remains the most sensitive method for diagnosing occult infection in a low-endemic European setting.

## 5. Conclusions

In conclusion, OBI was detected in a small but clinically relevant proportion of HBsAg-negative patients undergoing liver biopsy and may coexist with other causes of liver diseases. Because OBI was exclusively observed in anti-HBc-positive patients, these findings support total anti-HBc as the most useful screening marker for identifying patients in whom OBI may be present, although confirmation still requires detection of HBV DNA in liver tissue. These findings should be interpreted cautiously, and further multicenter studies with larger cohorts and longitudinal follow-up are needed to better define the clinical significance and long-term outcomes of OBI in different liver disease populations.

## Figures and Tables

**Figure 1:**
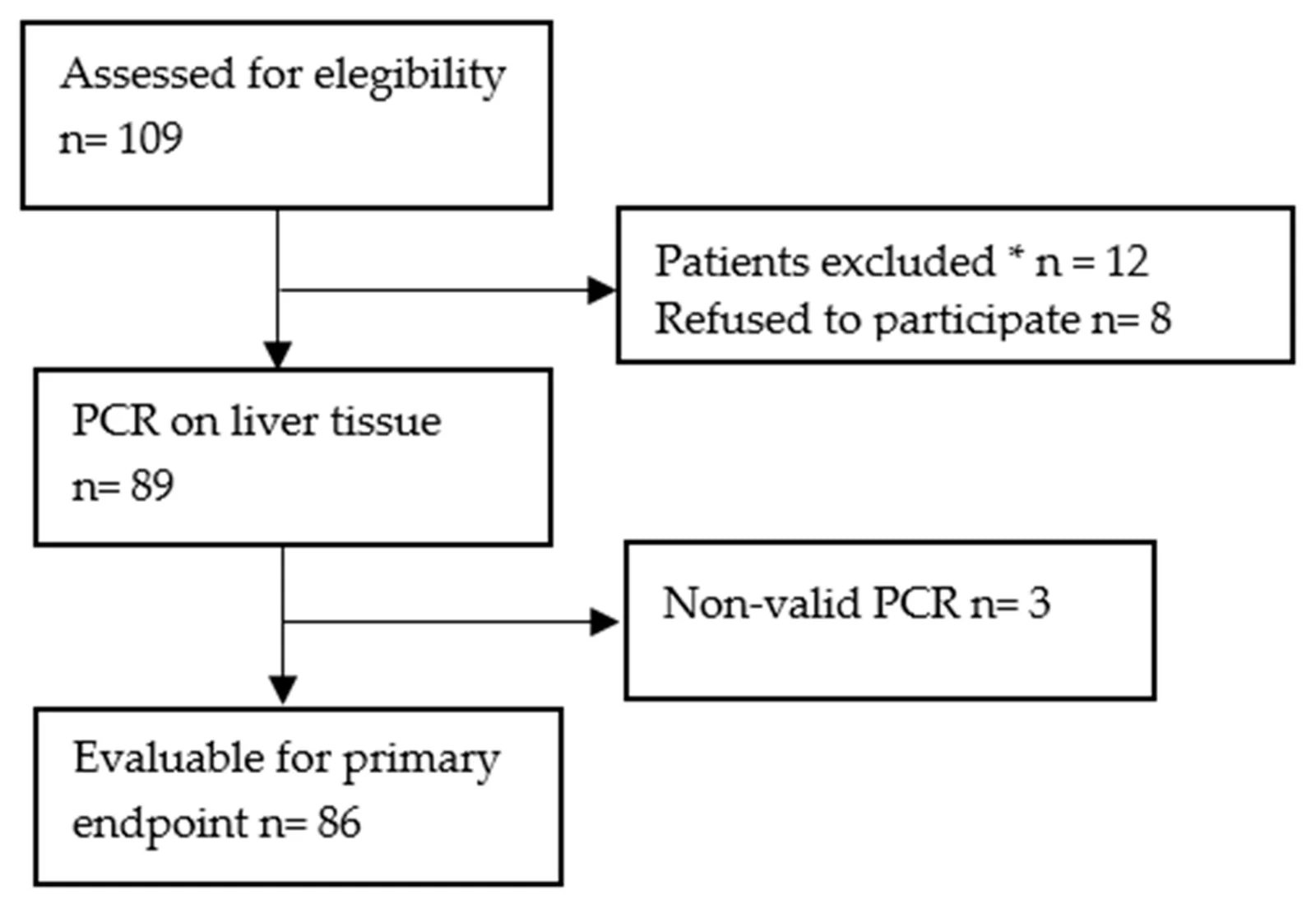
Flowchart of the study * HbsAg-positive n = 9, Hepatocellular carcinoma n = 3. Abbreviations: PCR, polymerase chain reaction.

**Table 1:** Baseline characteristics of patients included in the study.

	Total n = 86
Age (Mean ± SD)	49.8 ± 11.6
Gender (female) (n, %)	46 (53.5)
Caucasian (n, %)	79 (91.9)
Active alcohol intake (n, %)	11 (12.8)
Etiology liver disease (n, %) HCV MAFLD AIH PBC Alcohol Cryptogenic	25 (29.1) 22 (25.6) 20 (23.3) 13 (15.1) 5 (5.8) 1 (1.2)
Liver cirrhosis (n, %)	9 (10.5)
Elastography (KPa) (Mean ± SD)	6.7 ± 4.2
Liver steatosis by US (n, %)	25 (29.1)
Serum anti-HBc (n, %)	13 (15.1)
Serum anti-HBs (n, %)	15 (17.4)
Serum anti-HBc + antiHBs (n, %)	6 (7.0)
Documented HBV vaccination (n, %)	10 (11.6)

Abbreviations: AIH, autoimmune hepatitis; anti-HBc: hepatitis B core antibody; anti-HBs: hepatitis B surface antibody; HCV, hepatitis C; KPa, Kilopascal; MASLD, metabolic dysfunction-associated steatotic liver disease; PBC, primary biliary cholangitis; US: ultrasound.

**Table 2:** Characteristics of patients with OBI.

Patient	Age	Sex	Etiology	Liver Cirrhosis	Anti-HBc	Anti-HBs	Serum HBV DNA (IU/mL)
1	42	F	PBC	No	+	+	Undetectable
2	55	M	HCV	No	+	−	Undetectable
3	48	F	AIH	No	+	+	Undetectable
4	52	M	MASLD	No	+	−	25

Abbreviations: AIH, autoimmune hepatitis; anti-HBc, hepatitis B core antibody; anti-HBs, hepatitis B surface antibody; F, female; HCV, hepatitis C virus; M, male; MASLD, metabolic dysfunction-associated steatotic liver disease; PBC, primary biliary cholangitis.

## Data Availability

The data are not publicly available due to privacy and ethical restrictions concerning patient data.
